# Anti-neutrophil Cytoplasmic Antibody-Negative Rapid Progressive Glomerulonephritis With Mild Pathological Presentation in an Older Patient: A Case Report

**DOI:** 10.7759/cureus.61390

**Published:** 2024-05-30

**Authors:** Ryuichi Ohta, Keita Inoue, Chiaki Sano

**Affiliations:** 1 Communiy Care, Unnan City Hospital, Unnan, JPN; 2 Urology, Unnan City Hospital, Unnan, JPN; 3 Community Medicine Management, Shimane University, Izumo, JPN

**Keywords:** general medicine, family medicine, rural, renal insufficiency, hemodialysis, rituximab, cyclophosphamide, anca-negative vasculitis, rapidly progressive glomerulonephritis

## Abstract

This case report discusses the management of anti-neutrophil cytoplasmic antibodies (ANCA)-negative rapid progressive glomerulonephritis (RPGN) in a 68-year-old man with a complex medical history, presenting with fatigue, edema, and acute renal failure. Despite the absence of positive biomarkers for specific RPGN types, the clinical progression suggested microscopic polyangiitis, leading to intensive immunosuppressive therapy with cyclophosphamide and rituximab. The patient's condition was further complicated by the coexistence of nephritic and nephrotic syndromes, requiring nuanced management strategies, including prolonged hemodialysis. After initial treatment failure, remission was eventually achieved, allowing cessation of dialysis and significant recovery of renal function. This case highlights the challenges of diagnosing and managing ANCA-negative RPGN, particularly the importance of a tailored, dynamic approach to treatment in resource-limited settings. The recovery observed underscores the potential for renal function improvement even after prolonged periods of intensive therapy, reinforcing the need for persistence and adaptability in managing complex RPGN cases.

## Introduction

Rapid progressive glomerulonephritis (RPGN) is a critical renal disease that causes significant morbidity and mortality, necessitating immediate intervention to prevent irreversible kidney damage and dependency on hemodialysis [1]. The disease's varied etiology underscores the importance of prompt treatment initiation, even before the complete assessment of biomarkers and renal biopsy results [2]. The anti-neutrophil cytoplasmic antibodies (ANCA) biomarker plays a crucial role in diagnosing and managing RPGN, guiding clinicians in the prognosis and intensifying treatment with cyclophosphamide and rituximab in ANCA-positive cases [3,4].

However, managing ANCA-negative RPGN presents a considerable challenge, especially in settings with limited access to diagnostic resources like renal biopsies, often seen in rural areas [5,6]. This time, we experienced a case of ANCA negative RPGN in an older man. Based on the rapid clinical course, we suspected microscopic polyangiitis and used cyclophosphamide and rituximab intensively, leading to remission and avoiding constant hemodialysis. In addition, the treatment process was complicated due to the mixture of nephritic and nephrotic syndromes, needing nuanced treatments and prolonged hemodialysis. This case underscores the difficulties in diagnosing ANCA-negative RPGN and the importance of basing treatment decisions on the clinical course. It emphasizes the critical need for early and decisive intervention and waiting time for remission of RPGN from acute to chronic phrases, particularly in resource-limited settings, to achieve positive patient outcomes.

## Case presentation

A 68-year-old man came to a rural community hospital with chief complaints of fatigue, bilateral lower leg edema, watery diarrhea, and dyspnea. One month before admission, he noticed bilateral edema of the lower legs. Severe days before admission, the patient had fatigue and exacerbation of the edema of lower legs progressing to ankles to thighs. At the same time, he began to have watery diarrhea several times a day without abdominal pain. On the day of admission, he felt dyspnea in the night and came to the hospital. He did not have headaches, fever, rash, joint pain, other infectious symptoms, or travel histories to other countries. The past medical histories were hypertension and dyslipidemia. The medications were lisinopril 5 mg daily and atorvastatin 5 mg daily. He smoked one pack a day for 30 years and drank alcohol daily for 40 years.

The vital signs at the visit were as follows: blood pressure, 167/97 mmHg; pulse rate, 65 beats/min; body temperature, 36.8°C; respiratory rate, 16 breaths/min; and oxygen saturation, 98% on room air. The patient was alert to time, place, and person. Physical examination showed slow pitting edema of bilateral lower legs without petechiae and purpura. No other abnormal neurological findings were noted. There were no apparent abnormalities in the chest or abdomen and no skin eruptions. The laboratory tests showed a high creatinine and blood urea nitrogen (BUN) level and a low serum albumin level with proteinuria, hematuria, and granular casts (Table 1).

**Table 1:** Initial laboratory data of the patient eGFR, estimated glomerular filtration rate; CK, creatine kinase; CRP, C-reactive protein; TSH, thyroid-stimulating hormone; HCV, hepatitis C virus; SARS-CoV-2, severe acute respiratory syndrome coronavirus 2; HBs, hepatitis B surface antigen; HBc, hepatitis B core antigen; C3, complement component 3; C4, complement component 4; S/CO, sample/cut off; LPF, low power field; BUN, blood urea nitrogen

Parameter	Level	Reference
White blood cells	10.50	3.5-9.1×10^3^/μL
Neutrophils	82.7	44.0-72.0%
Lymphocytes	9.9	18.0-59.0%
Monocytes	6.2	0.0-12.0%
Eosinophils	0.6	0.0-10.0%
Basophils	0.6	0.0-3.0%
Red blood cells	5.02	3.76-5.50×10^6^/μL
Hemoglobin	16.2	11.3-15.2 g/dL
Hematocrit	46.7	33.4-44.9%
Mean corpuscular volume	92.9	79.0-100.0 fL
Platelets	24.4	13.0-36.9×10^4^/μL
Erythrocyte sedimentation rate	82	2-10 mm/hour
Total protein	4.5	6.5-8.3 g/dL
Albumin	1.5	3.8-5.3 g/dL
Total bilirubin	0.3	0.2-1.2 mg/dL
Aspartate aminotransferase	20	8-38 IU/L
Alanine aminotransferase	11	4-43 IU/L
Alkaline phosphatase	98	106-322 U/L
γ-Glutamyl transpeptidase	36	<48 IU/L
Lactate dehydrogenase	356	121-245 U/L
BUN	51.8	8-20 mg/dL
Creatinine	1.76	0.40-1.10 mg/dL
eGFR	31.1	>60.0 mL/min/1.73m^2^
Serum Na	142	135-150 mEq/L
Serum K	3.8	3.5-5.3 mEq/L
Serum Cl	108	98-110 mEq/L
Serum Ca	7.0	8.8-10.2 mg/dL
Serum P	3.8	2.7-4.6 mg/dL
Serum Mg	1.9	1.8-2.3 mg/dL
CK	36	56-244 U/L
CRP	3.02	<0.30 mg/dL
TSH	2.77	0.35-4.94 μIU/mL
Free T4	0.7	0.70-1.48 ng/dL
IgG	601	870-1700 mg/dL
IgM	56	35-220 mg/dL
IgA	351	110-410 mg/dL
IgE	135	<173 mg/dL
HBs antigen	0.0	IU/mL
HBs antibody	0.67	mIU/mL
HBc antibody	0.00	S/CO
HCV antibody	0.00	S/CO
Syphilis treponema antibody	0.00	S/CO
SARS-CoV-2 antigen	Negative	Negative
Anti-nuclear antibody	40	<40
Homogeneous	40	<40
Speckled	40	<40
C3	177	86-164 mg/dL
C4	61	17-45 mg/dL
Urine test	-	-
Leukocyte	Negative	Negative
Nitrite	Negative	Negative
Protein	(4+)	Negative
Glucose	Negative	Negative
Urobilinogen	Negative	Negative
Bilirubin	Negative	Negative
Ketone	Negative	Negative
Blood	(2+)	Negative
pH	6.0	-
Specific gravity	1.050	-
Granule cast	>10/LPF	Negative
Epithelial cast	>10/LPF	Negative
24-hour urinary protein excretions	17.41 g/day	Negative

**Figure 1:**
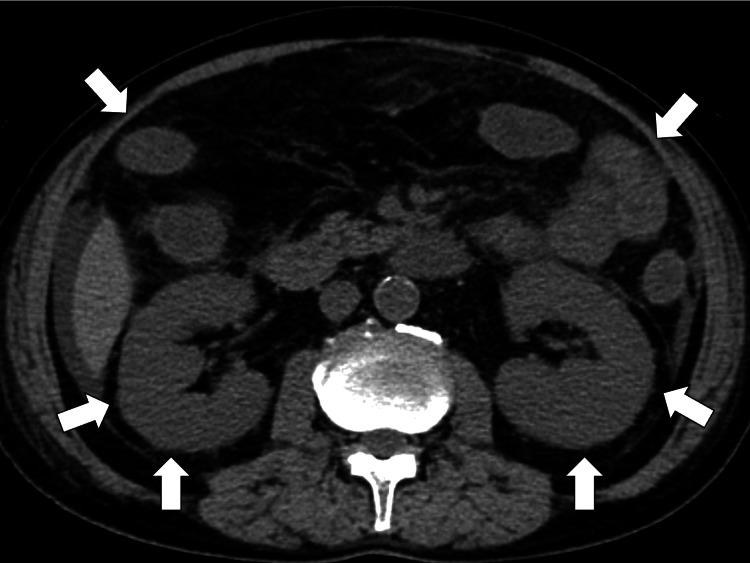
Abdominal CT showing the swelling of bilateral kidneys and diffuse edematous change of the small intestine and colon (white arrows) CT, computed tomography

Abdominal computed tomography (CT) showed the swelling of bilateral kidneys and diffuse edematous change of the small intestine and colon (Figure 1).

Based on the clinical findings, the patient was diagnosed tentatively with RPGN, complicated with nephritic and nephrotic syndrome. The patient was treated with methylprednisolone 1000 mg intravenously for three days, followed by prednisolone 60 mg orally. Three days later, the additional laboratory data investigating the etiologies of RPGN showed negative results of anti-nuclear antibodies, ANCA, and anti-glomerular basement membrane antibodies. The same day, the renal biopsy was performed for further investigation. The patient’s creatinine and BUN elevated progressively, so intravenous cyclophosphamide of 500 mg was used for the remission of RPGN symptoms. 

**Figure 2:**
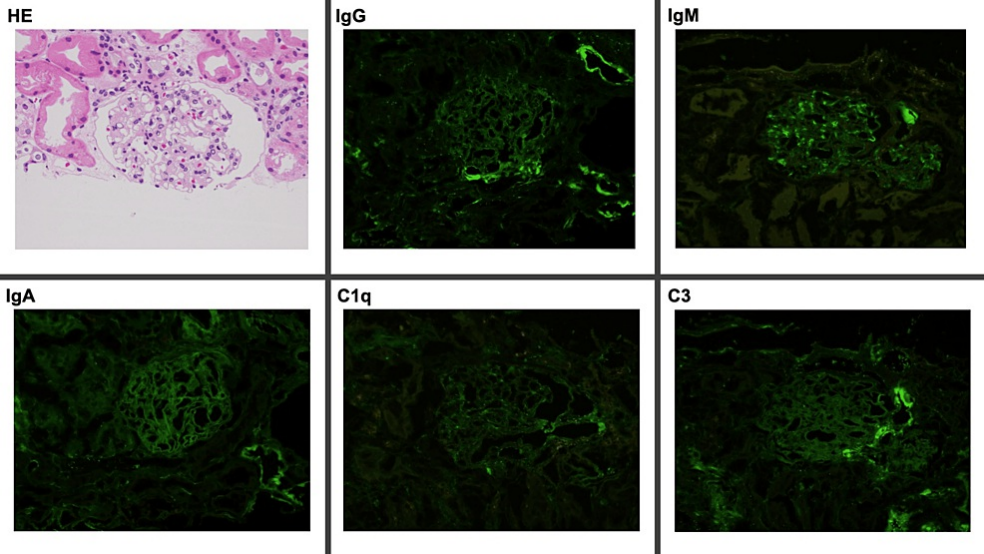
The pathological findings of the kidneys showing mild infiltration of Immunoglobin M and G without any crescent formation C, complement; HE, hematoxylin & eosin

In the initial week of the admission, the patient’s diarrhea disappeared. Still, edema on the extremities was progressive, and his creatine and BUN increased gradually to creatinine levels of 6.62 mg/dL and 136 mg/dL. His dyspnea was exacerbated, and emergent hemodialysis was performed. The pathological findings of the kidneys showed mild infiltration of Immunoglobin M and G without any crescent formation (Figure 2).

Based on the progressiveness of RPGN, the possibility of ANCA-negative microscopic polyangiitis was considered. On day 11, through a discussion with the patient and families, rituximab of 500 mg/week for four weeks was started. 

After the remission of systemic inflammation from RPGN, considering a lower level of C-reactive protein (CRP), the persistency of urinary casts and proteinuria from nephritic and nephrotic syndrome continued and caused intravascular hypovolemic status, leading to persistent prerenal kidney injury. For the increase in the colloid osmotic pressure in vessels, intravenous albumin infusion started from day 10 of admission for five days. In addition, to increase the urine output, furosemide of 100 mg per day was started intravenously. However, on day 21 of the admission, his urine output completely stopped and depended on hemodialysis.

His treatment for RPGN was continued with hemodialysis and tapering prednisolone every two weeks. In addition, systemic management such as blood glucose level, blood pressure, and anemia continued through calorie intake control, anti-hypertensive medication (lisinopril 10 mg and amlodipine 10 mg daily), and hypoxia-inducible factor prolyl-hydroxylase inhibitor (daprodustat 6 mg daily). On day 52, his urine output restarted from 40 to 100 mL/day and gradually increased the following days. By day 60 of admission, his urine output increased by 1000 mL/day, and his hemodialysis was ended. On day 72 of admission, his renal functions improved to serum creatinine of 1.02 mg/dL and BUN of 21 mg/dL (estimated glomerular filtration rate of 49.2 mL/min/1.73 m^2^). He was discharged to his home with his usual lifestyle. His CRP fluctuated from 0.3 mg/dL to 2.1 mg/dL in outpatient department follow-up with no change of renal function.

## Discussion

The management of ANCA-negative RPGN remains a formidable challenge, demanding a nuanced understanding of its pathophysiology and a strategic approach to treatment. As this case shows, the criticality of timely and aggressive treatment should be balanced, given the disease's potential for rapid progression to end-stage renal disease (ESRD) without appropriate intervention. In addition, as the initial intensive treatments are ineffective, the delay in the recovery of renal functions should be considered in the next few months. This case emphasizes the importance of a multifaceted treatment strategy that is dynamic and responsive to the patient's evolving clinical condition and the possibility of delayed recovery of renal functions in ANCA-RPGN.

The essence of treating ANCA-negative RPGN involves the integration of immunosuppressants and biological agents tailored to the individual patient's disease activity and clinical presentation [7]. The rationale behind this approach is grounded in the understanding that the pathogenesis of ANCA-negative RPGN, while still fully elucidated, involves an aberrant immune response leading to glomerular injury [8]. As this case shows, immunosuppressants, such as corticosteroids and cyclophosphamide, have been foundational in inducing remission, particularly in the early stages of the disease [6]. However, the advent of biological therapies offers a targeted approach to modulate specific pathways of the immune system involved in the disease process, potentially offering a more favorable side effect profile and improved outcomes to particular subsets of patients [9]. In this case, considering the progression of RPGN, cyclophosphamide and rituximab were used consecutively, eventually remitting the inflammation of ANCA-negative RPGN. Even if the initial pathological findings are mild, the speed of the progression of ANCA-negative RPGN demands intensive treatments through clinically assessing patients' conditions [10].

An essential advancement in the management of ANCA-negative RPGN is the emerging focus on the changes in laboratory and urinary findings, focusing on various clinical courses with a wide range of recovery [11]. This approach seeks to optimize therapeutic efficacy while minimizing toxicity, which is particularly important in a narrow therapeutic window [11]. For example, the use of rituximab, a monoclonal antibody targeting CD20+ B cells, has shown promise in some instances of RPGN where traditional therapies have failed [12]. However, the appearance of signs of recovery varies, regardless of patients' backgrounds and treatment [13]. As this case shows, patients' recovery could start two months after the initial intensive therapies. Predicting clinical courses in RPGN necessitates ongoing research and clinical trials to better understand the disease's molecular underpinnings and identify novel therapeutic targets [14]. General physicians should not give up on the possibility of recovering the renal function of ANCA-negative RPGN, even if the initial intensive treatments show no effects in several weeks [15,16].

The dynamic nature of RPGN, with potential clinical presentation and disease activity fluctuations, underscores the necessity for continuous monitoring and treatment adjustment. This iterative process involves regular assessment of renal function, proteinuria, hematuria, and other relevant biomarkers alongside clinical indicators of disease activity [17]. However, as this case shows, the prediction of the clinical courses from initial presentations may need to be corrected and may be challenged by various exacerbations. The coexistence of nephritic and nephrotic syndromes within the spectrum of RPGN poses particular challenges, necessitating an adjusted approach to management [18]. The differentiation between these syndromes is crucial, as it influences the choice of therapeutic agents and the management of complications [18]. For instance, the presence of significant proteinuria and hypoalbuminemia in nephrotic syndrome may warrant the use of angiotensin-converting enzyme inhibitors or angiotensin receptor blockers to reduce proteinuria, alongside specific measures to address the increased risk of thromboembolism and hyperlipidemia. As this case shows, as system-specific specialists, general physicians should monitor systemic findings such as blood pressure, anemia, and glucose levels and control each clinical finding continually, waiting for signs of recovery for several months [19]. 

## Conclusions

In this case of ANCA-negative RPGN, the management challenges underscore the necessity for early and aggressive interventions to forestall ESRD, even in the absence of specific biomarkers. Our approach, utilizing cyclophosphamide and rituximab based on clinical progression rather than initial laboratory findings, highlights the critical role of dynamic treatment strategies in achieving remission. Despite the patient's complex presentation and prolonged dependency on dialysis, the eventual recovery of renal function emphasizes the importance of adaptability in treatment plans and continuous monitoring. This case reinforces the imperative for ongoing research and innovative therapeutic strategies to enhance outcomes in managing RPGN, particularly in resource-limited settings.
